# Neutrophil extracellular traps amplify neutrophil recruitment and inflammation in neutrophilic asthma by stimulating the airway epithelial cells to activate the TLR4/ NF-κB pathway and secrete chemokines

**DOI:** 10.18632/aging.103479

**Published:** 2020-08-05

**Authors:** Rongjun Wan, Juan Jiang, Chengping Hu, Xi Chen, Cen Chen, Bingrong Zhao, Xinyue Hu, Zhiyuan Zheng, Yuanyuan Li

**Affiliations:** 1 Department of Respiratory Medicine, National Key Clinical Specialty, Branch of National Clinical Research Center for Respiratory Disease, Xiangya Hospital, Central South University, Changsha, Hunan, China; 2 Hunan Provincial Clinical Research Center for Respiratory Diseases, Xiangya Hospital, Changsha, Hunan, China; 3 National Clinical Research Center for Geriatric Disorders, Xiangya Hospital, Changsha, Hunan, China

**Keywords:** neutrophilic asthma, neutrophil extracellular traps, chemotaxis, airway inflammation

## Abstract

Neutrophilic asthma (NA) is a distinct airway inflammation disease with prominent neutrophil infiltration. The role played by neutrophil extracellular traps (NETs) in NA, however, is quite unclear. This study was based on the hypothesis that NETs are responsible for the second neutrophil wave and therefore contribute significantly to inflammation. The proinflammatory effects of NETs were evaluated in vitro and in vivo. Formation of NETs and neutrophil swarming was seen in a mouse model of NA. Additionally, NETs were found to stimulate airway cells to express CXCL1, CXCL2, and CXCL8 via the TLR4/NF-κB pathway, which recruits neutrophils to the inflammation site. Furthermore, prevention of NET formation decreased the recruitment of lung neutrophils and hence reduce neutrophilic inflammation. Additionally, the structural integrity of NETs had no effect on the recruitment of lung neutrophils and neutrophilic inflammation. In NA mice, NETs could trigger airway and alveolar epithelial cells to express chemokines which recruit more neutrophils via activation of the TLR4/NF-κB pathway.

## INTRODUCTION

Asthma is an inflammatory disease characterized by airway hyper responsiveness [1]. According to the proportion of different inflammatory cells in the bronchial alveolar lavage fluid (BALF), asthma can be divided into eosinophilic, neutrophilic (NA), mixed-granulocytic, and paucigranulocytic asthma [2]. NA is defined by the presence of more than 65% neutrophils and less than 3% eosinophils in induced sputum [2, 3]. Clinically, patients with NA suffer very severe symptoms and are glucocorticoid resistant [2, 4].

Neutrophils are the most abundant innate immune cells in peripheral blood and are the body's first line of defense against pathogen invasion. In NA, neutrophil swarming is a neutrophilic accumulative process that consists of two waves. The first wave is recruited to airways by stimulation by antigen or by another stimuli. Neutrophils promote airway gland hypersecretion of matrix metalloproteinase-9 and neutrophil elastase, which contribute to airway wall remodeling [5]. Additionally, they also stimulate airway epithelial cells to secrete potent chemoattractants such as CXCL8, thereby recruiting more neutrophils resulting in a self-sustained vicious cycle in the airways [6]. The first wave of neutrophils interacts with the airway cells to amplify the second wave, thus aggravating neutrophilic inflammation [7, 8]. However, the regulatory mechanisms that cause the first wave to result in the second neutrophil wave remain unknown.

Neutrophils can produce neutrophil extracellular traps (NETs) in acute inflammation. This is mediated by a programmed cell death process that differs from apoptosis and necrosis and has therefore been recognized as an additional mechanism of neutrophil defense [9–11]. NETs form a mesh-like structure that consists of dsDNA and neutrophilic proteinases, including neutrophil elastase (NE) and myeloperoxidase (MPO) [9, 12]. Once released into the extracellular space, NETs participate in innate immunity against pathogens [9]. NETs, however, are known as a double-edged sword; while controlled NET formation assists the immune system in fighting against pathogens, exaggerated NET formation can aggravate inflammatory reactions and damage tissue [7, 13]. NETs and inflammatory cytokines are known to be elevated in the BALF of NA patients. The concentration of cytokines produced by NETs is known to be positively correlated to the concentration of inflammatory cytokines and the severity of the disease [7, 14, 15]. Despite a recent surge in research into the role of NETs in NA pathogenesis, the mechanism of their involvement in the second wave of the neutrophil swarming by stimulation of the airway cells is still unknown.

Neutrophils react to multiple endogenous and exogenous chemoattractants, such as the four kinds of neutrophilic chemoattractants namely: lipids, N-formylated peptides, complement anaphylatoxins, and chemokines [16]. Chemokines with the glutamate-leucine-arginine (ELR) motif before a CXC motif (ELR-CXC chemokines) are a series of chemokines that have the ability to promote neutrophil migration; CXCL1 (GRO-α), CXCL2 (GRO-β), and CXCL8 (IL-8) are three examples of common ELR-CXC chemokines [16, 17]. They activate CXC chemokine receptor (CXCR) 1 or 2, attracting neutrophils in a concentration gradient manner and guiding them to the inflammatory site for further reactions [15]. Additionally, previous studies indicate that these chemokines are known to be expressed by epithelial cells and endothelial cells [16, 18].

This study therefore hypothesized that NETs recruit large numbers of neutrophils by stimulating human bronchial epithelial cells (HBEs) and human alveolar epithelial cells (HAEs) to express chemokines. The NETs formed by the airway neutrophils of NA mice were investigated along with the effect of these NETs on chemokine expression and neutrophil recruitment. Furthermore, this study investigated the in vitro effect of neutrophilic chemokines produced by HBEs and A549 on neutrophilic migration following the stimulation of NETs. By elucidating the mechanism by which the first wave of neutrophils recruits the second wave, we hope to find intervention targets and therefore drugs to provide new directions for the treatment of neutrophilic asthma.

## RESULTS

### NETs are generated in a mouse model of NA

**Figure 1:**
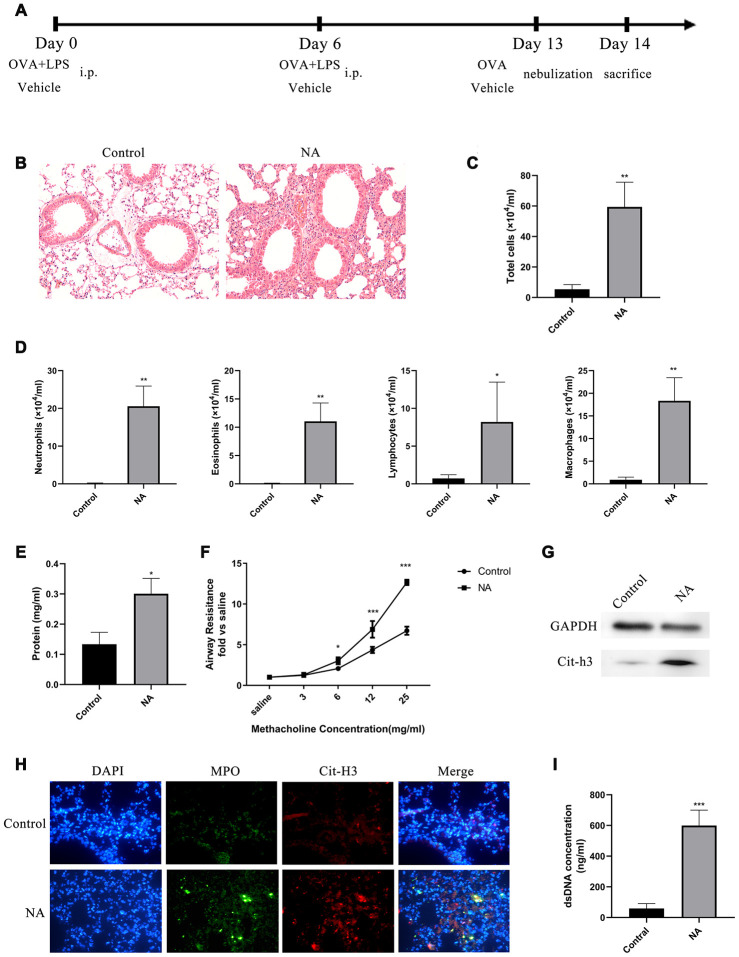
**NETs were generated in a mouse model of neutrophilic asthma (NA).** (**A**) Flow chart for generation of NA mice. (**B**) HE staining of lungs showed prominent inflammatory cell infiltration around the bronchi and blood vessels of NA mice (200X). (**C**–**E**) Total cells, differential cell count, and total protein in the BALF supernatant of NA mice were measured. (**F**) Airway resistance was measured in NA mice after treatment with different concentrations of methacholine. (**G**) Cit-H3 expression was measured by western blotting. (**H**) Cit-H3 and MPO expression was measured by immunofluorescence (400X). (**I**) dsDNA concentration in the BALF supernatant of NA mice was detected by PicoGreen analysis. *: P<0.05, **: P<0.01, ***: P<0.001 vs control group.

A mouse model of NA was generated to investigate the role of NETs in the aggravation of neutrophil recruitment and inflammation (Figure 1A). HE staining showed that robust neutrophil inflammation was located around the bronchovascular bundle of the NA mice (Figure 1B). Total cell count, especially of neutrophils, and total protein in the BALF of NA mice were significantly increased (Figure 1C–1E). Compared to the control group, airway resistance was significantly increased in NA mice after induction with a higher dose of methacholine (Mch) nebulization (Figure 1F). Western blotting and immunofluorescence showed that specific NET markers, Cit-H3 and MPO, were highly expressed in this mouse model of NA (Figure 1G and 1H). Additionally, dsDNA levels in BALF of NA mice were robustly higher than in the control group (Figure 1I). Thus, the results showed that higher inflammation levels, neutrophil counts, and NETs existed in the airway of NA mouse model.

### Preventing NET formation can alleviate lung neutrophilic inflammation, without the structural integrity of NETs having any effect

**Figure 2:**
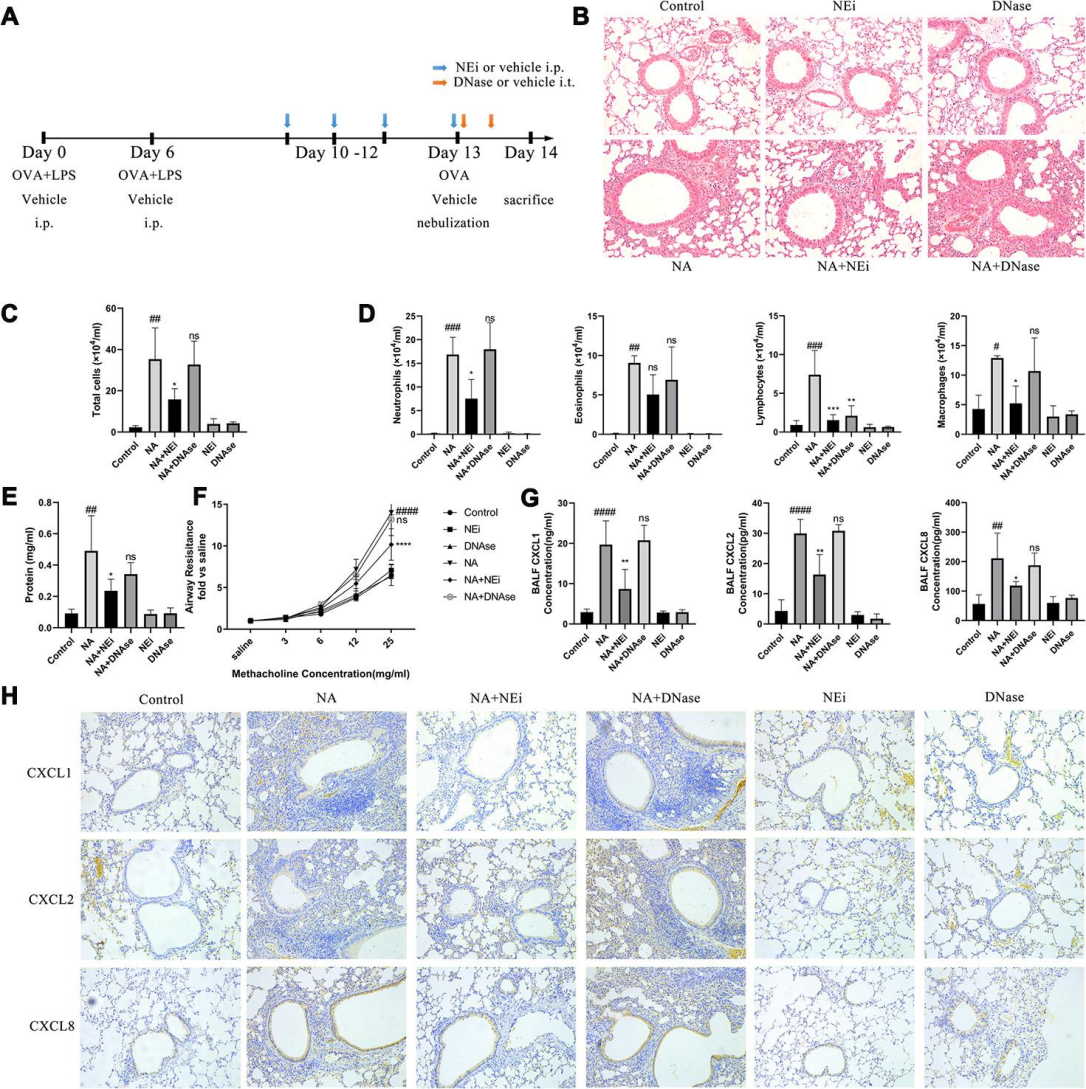
**Inhibition of NET formation alleviated neutrophilic airway inflammation in NA model, rather than degrading the NETs DNA.** (**A**) Flow chart for sivelestat or DNase I administration intraperitoneally. (**B**) HE staining results showed that sivelestat could alleviate inflammation while DNase I could not (200X). (**C**–**E**) Total cells, differential count of cells, and total protein were measured after treatment with sivelestat or DNase I in the BALF of NA mice. (**F**) Airway resistance was measured after methacholine treatment. (**G**) Concentration of CXCL1, CXCL2 and CXCL8 in the BALF supernatant of NA mice was measured by ELISA. (**H**) CXCL1, CXCL2, and CXCL8 expression was measured by Immunohistochemistry (200X). #: P<0.05, ##: P<0.01, ###: P<0.001, ####: P<0.0001 vs control group. *: P<0.05, **: P<0.01, ***: P<0.001, ****: P<0.0001 vs NA group. ns = no statistical difference vs NA group.

The NA mice were then treated with sivelestat (selective neutrophil elastase inhibitor) and DNase I to evaluate the role played by NETs in neutrophilic airway inflammation (Figure 2A). Sivelestat was added to prevent NET formation. DNase I was added to deconstruct the backbone of NETs in the NA mice. HE stain results showed that sivelestat treatment prevented neutrophil recruitment, relieved airway inflammation, reduced the BALF cells and neutrophil count, increased the BALF total protein content, and alleviated airway resistance, while DNase I treatment had no effect on these (Figure 2B–2F). It was also confirmed that sivelestat and DNase I were both able to eliminate NETs according to the immunofluorescence, western blotting, and BALF PicoGreen analysis results (Supplementary Figure 1). Furthermore, the expression of CXCL1, CXCL2, and CXCL8 was significantly increased in NA mice as compared to control mice, but declined after sivelestat treatment; DNase I treatment had no obvious effect in the NA mice (Figure 2G–2H). It is worth noting that chemokines were mainly expressed in HBEs and HAEs (Figure 2H). These results indicate that NETs promote airway inflammation in the NA model independently of their structural integrity.

### The TLR4/NF-κB pathway is activated in this NA model and is responsible for airway inflammation

**Figure 3:**
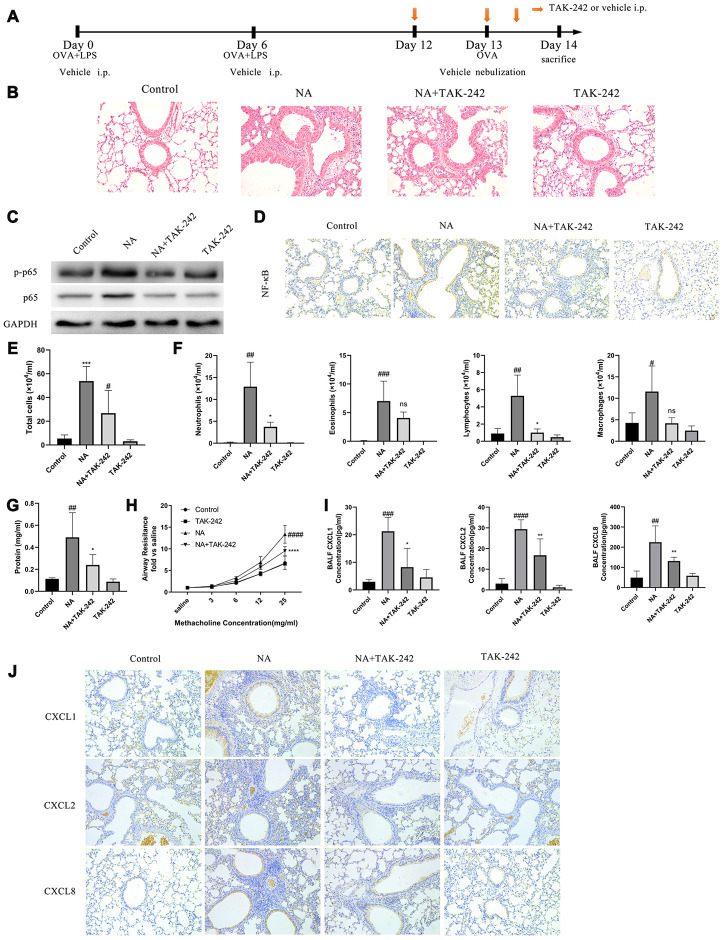
**Blocking the TLR4/NF-κB pathway with TAK-242 reduced the expression of chemotactic factors and alleviate airway inflammation in NA mice.** (**A**) Flow chart for intraperitoneal administration of TAK-242. (**B**) HE staining results showed that TAK-242 treatment could alleviate inflammation (200X). (**C**) p65 NF-κB and p-p65 NF-κB expression was measured by western blotting. (**D**) p65 NF-κB expression was measured by immunohistochemistry (200X). (**E**–**G**) Total cells, differential cells, and total protein were measured in the BALF of NA mice. (**H**) Airway resistance was measured after methacholine treatment. (**I**) CXCL1, CXCL2, and CXCL8 expression in the BALF of NA mice was measured by ELISA. (**J**) CXCL1, CXCL2, and CXCL8 expression was measured by Immunohistochemistry in the lung tissues of NA mice. #: P<0.05, ##: P<0.01, ###: P<0.001, ####: P<0.0001 vs control group. *: P<0.05, **: P<0.01: ***: P<0.001, ****: P<0.0001 vs NA group. ns = no statistical difference vs NA group.

The role of NET-induced neutrophilic inflammation via TLR4/NF-κB pathway by treatment with TAK-242, a TLR4 inhibitor, was evaluated in NA mice (Figure 3A). The p-p65 NF-κB expression was found to be obviously increased with the transfer of NF-κB from cytoplasm to nucleus in the airway cells of NA mice, while TAK-242 treatment inhibited p-p65 NF-κB expression and NF-κB transfer (Figure 3C and 3D). Additionally, TAK-242 treatment significantly alleviated neutrophilic inflammation, reduced the BALF cells and neutrophil count, increased the BALF total protein content, alleviated airway resistance, and decreased the CXCL1, CXCL2, and CXCL8 expression (Figure 3B, 3E–3H, and Supplementary Figure 1). As expected, the TLR4/NF-κB pathway was activated in the airway epithelial cells of NA mice and was responsible for airway inflammation.

### NETs stimulate HBEs and A549 to express neutrophilic chemokines in vitro

**Figure 4:**
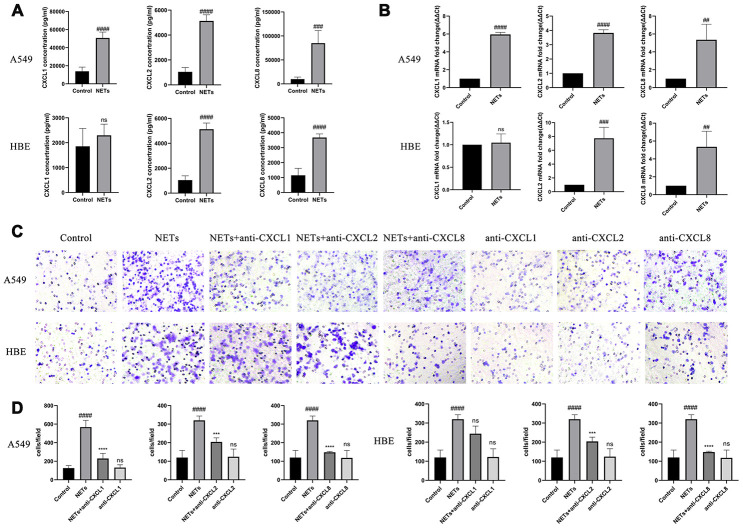
**NETs stimulate HBEs and A549 to express neutrophilic chemokines.** (**A** and **B**) CXCL1, CXCL2, and CXCL8 expression in the supernatant was analyzed by Luminex and RT-qPCR, after HBEs (or A549) were treated with NETs co-cultured for 24 h. (**C** and **D**) Neutrophil migration was analyzed by transwell after cell culture supernatant was induced for 48 h (200X). NET treated supernatant was added to the lower chamber. #: P<0.05, ##: P<0.01, ###: P<0.001, ####: P<0.0001 vs control group. *: P<0.05, **: P<0.01, ***: P<0.001, ****: P<0.0001 vs NA group. ns = no statistical difference vs NETs group.

Twenty-four hours after HBEs or A549 cells were treated with NETs, cell culture supernatant was collected for Luminex and transwell analyses. CXCL1, CXCL2, and CXCL8 expression was significantly increased in the HBE and A549 culture supernatants, with an exception for CXCL1 in HBEs, in the NETs treated group as compared to the control group (Figure 4A and 4B). Additionally, the cell culture supernatant in the NET-treated group was able to promote neutrophil migration, whereas the effect was reversed with the addition of the neutralizing CXCL1, CXCL2, and CXCL8 antibodies, especially neutralizing CXCL8 (Figure 4C and 4D). These results showed that airway epithelial cells could recruit neutrophils under stimulation of NETs.

### Degrading NETs by DNase I cannot eliminate chemokine stimulating effects in vitro

**Figure 5:**
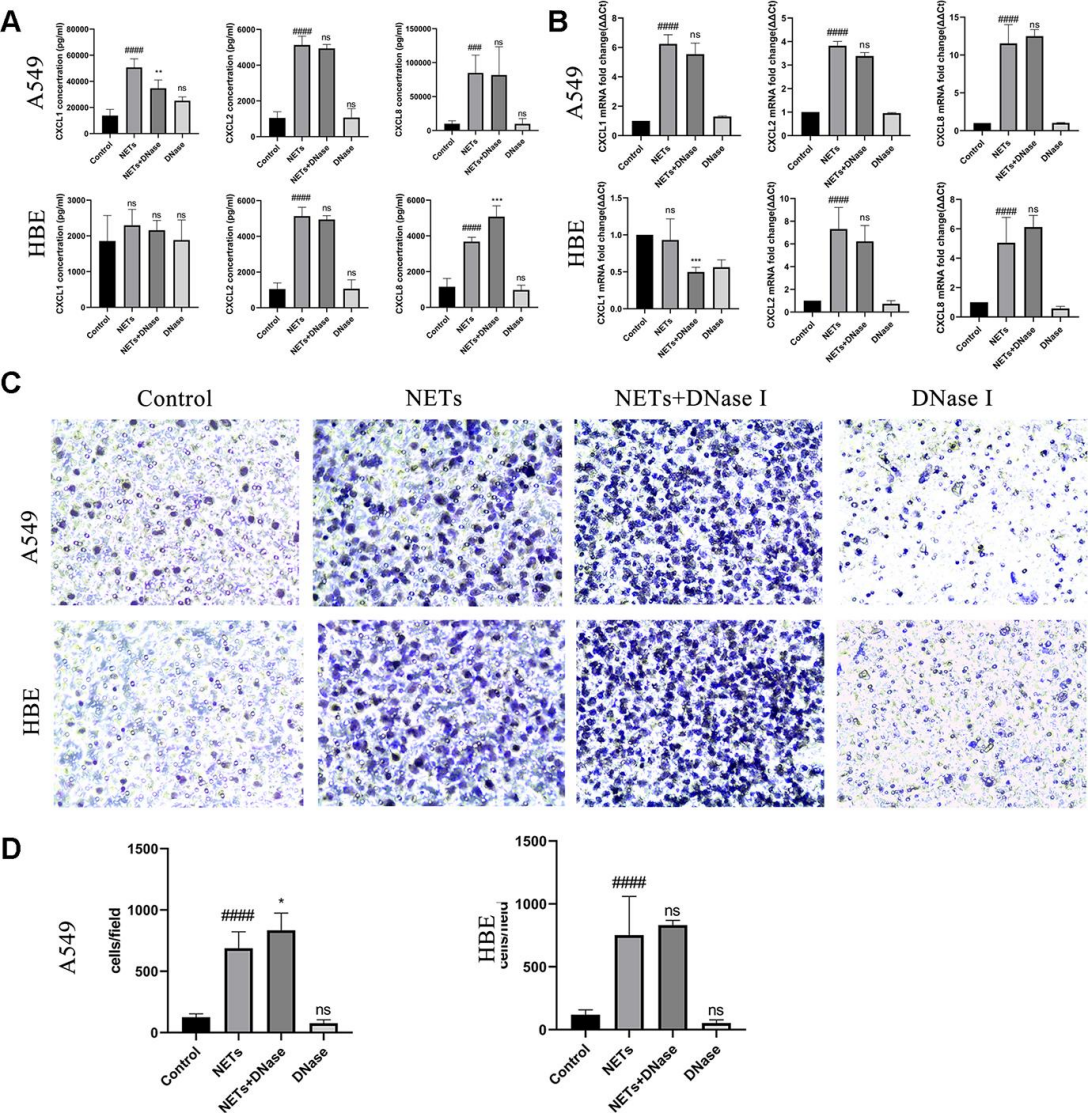
**Degradation of NETs by DNase I has no effect on chemokine expression and neutrophil migration in vitro.** (**A**, **B**) After treatment of HBEs and A549 cells with DNase I and NETs simultaneously, CXCL1, CXCL2, and CXCL8 levels in the supernatant were analyzed by Luminex and RT-qPCR. (**C** and **D**) After treatment of HBEs and A549 cells with DNase I and NETs simultaneously, the supernatant was collected and added into the lower chamber. Neutrophils were cultured in the upper chamber and neutrophil migration was analyzed by transwell after cell culture supernatant was induced for 48 h (200X). #: P<0.05, ##: P<0.01, ###: P<0.001, ####: P<0.0001 vs control group. *: P<0.05, **: P<0.01, ***: P<0.001: ****, P<0.0001 vs NA group. ns = no statistical difference vs NA or control group.

To assess the effect of NETs on the stimulation of airway epithelial cells expressing chemokines and recruiting neutrophils in vitro, NETs were generated by using 500 μM PMA to treat fresh neutrophils [19]. Next, the effect of in vitro degradation of the backbone structure of NETs was evaluated using DNase I which could reverse the chemokine stimulating effects of directly treated NETs. HBEs or A549 cells were treated with DNase I and NETs simultaneously as described previously [19]. Similar to the results obtained in the mice NA model, DNase I treatment had no effect on CXCL1, CXCL2, or CXCL8 expression or neutrophil migration (Figure 5). These results showed that destroying the structural integrity of NETs could not eliminate its stimulatory effects.

### Neutrophilic chemotaxis by stimulation of NETs via the TLR4/NF-κB pathway in vitro

**Figure 6:**
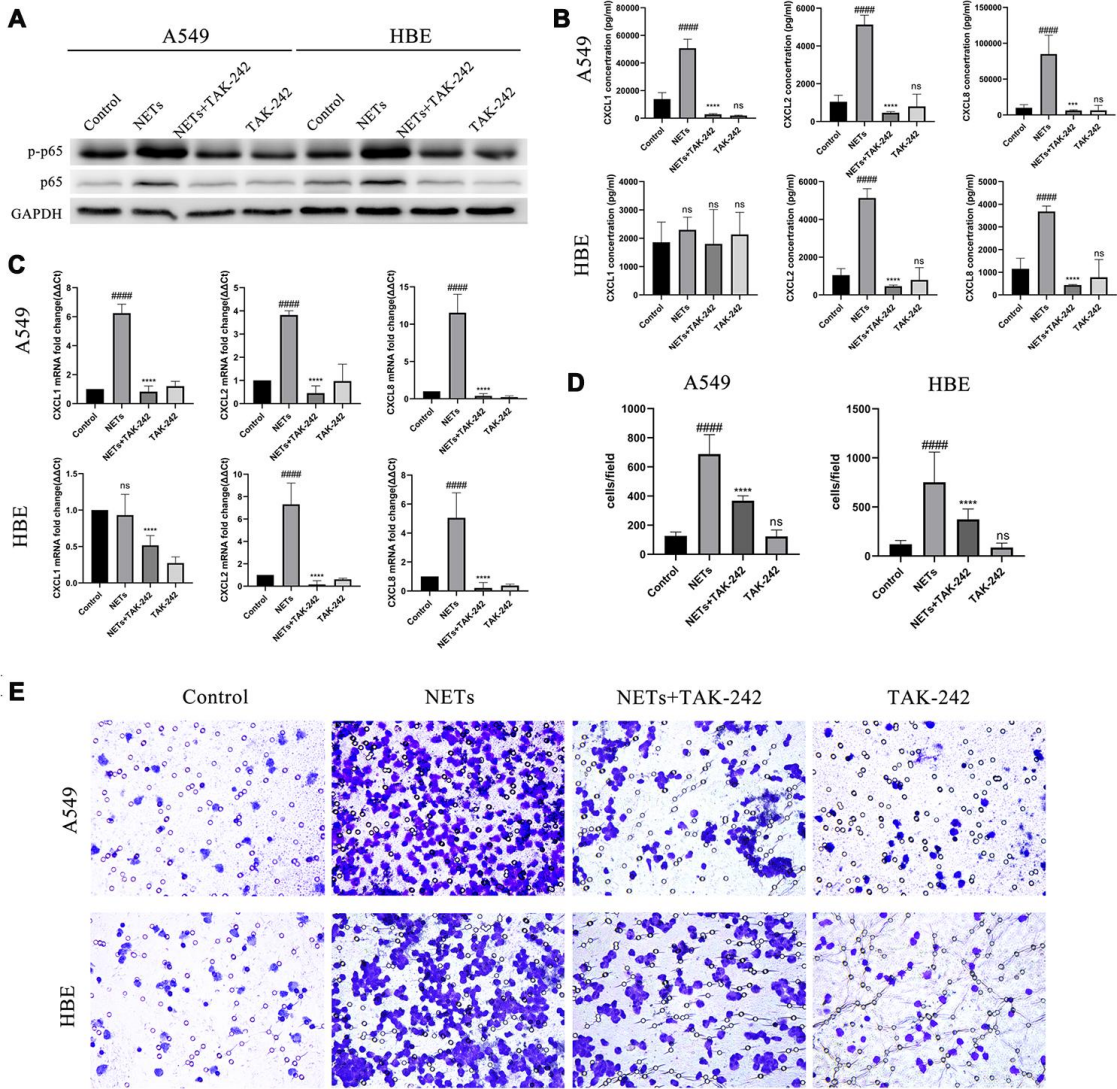
**Inhibition of the TLR4/NF-κB pathway reduced chemokine expression and neutrophil migration in vitro after stimulation of HBEs and A549 by NETs.** (**A**) After treatment of HBEs and A549 cells with TAK-242 and NETs simultaneously, p-p65 NF-κB and p65 NF-κB expression was measured by western blot. (**B** and **C**) After treatment of HBEs and A549 cells with TAK-242 and NETs simultaneously, CXCL1, CXCL2, and CXCL8 levels in the supernatant were analyzed by Luminex and RT-qPCR. (**D** and **E**) After treatment of HBEs and A549 cells with TAK-242 and NETs simultaneously, the supernatant was collected and added into the lower chamber. The neutrophils were then cultured in the upper chamber and neutrophil migration was analyzed by transwell after cell culture supernatant was induced for 48 h (200X). #: P<0.05, ##: P<0.01, ###: P<0.001, ####: P<0.0001 vs control group. *: P<0.05, **: P<0.01, ***: P<0.001, ****: P<0.0001 vs NA group. ns = no statistical difference vs NA or control group.

The direct activation of TLR4/NF-κb pathway by NETs in vitro was evaluated. HBEs and A549 cells were treated with TAK-242 and NETs simultaneously. After treatment for 48 h, p-p65 NF-κB expression was obviously inhibited in the combined TAK-242 and NETs groups but not in in the NETs group (Figure 6A). Additionally, CXCL1, CXCL2, and CXCL8 expression was significantly decreased in HBEs and A549s culture supernatants, with an exception for CXCL1 in HBEs, in the combined TAK-242 and NETs groups rather than in the NETs group (Figure 6B and 6C). Additionally, cell culture supernatants in both TAK-242 and NETs groups resulted in a significant reduction of neutrophil migration compared to the NETs group (Figure 6D and 6E).

## DISCUSSION

Neutrophils can produce NETs in acute inflammation via a programmed cell death process [9, 10]. Previous studies revealed that the first wave of neutrophils recruited further neutrophils to the inflammatory site depending on their interaction with other tissues and cells [17, 18]. In this study, NET formation and release were found to be involved in subsequent neutrophil recruitment in the NA mice.

A previous study found that NETs were present in the airways of patients with asthma and chronic obstructive pulmonary disease (COPD), which is associated with inflammation [20]. NET formation induced by cigarette smoke extract can promote the differentiation of Th1 and Th17 and initiate COPD [21], and it also induces the secretion of inflammatory cytokines such as IL-1β, IL-6, and TNF-α in HBEs [22]. In this study, we also found that higher inflammation, neutrophil count, and increased NET formation were present in the airways of NA mice. Additionally, we found that airway cells stimulated by NET supernatant can lead to neutrophil migration. This result showed that NETs stimulate airway cells to recruit the second wave of neutrophils.

Furthermore, we found that sivelestat treatment prevented neutrophil migration and recruitment, relieved airway inflammation, and alleviated airway resistance, whereas DNase I treatment caused no obvious improvement. Sivelestat is an NEi that prevents NET formation. DNase I administration was responsible for degradation of NET structure and eliminates or alleviates the cell toxic effects of NETs [23, 24]. However, a previous study and this study exhibited a very weak or noneffective result in any DNase I treatment in vivo or in vitro [25]. As previously described, DNA back-bone, neutrophil elastase, myeloperoxidase, and other proteinases are responsible for the formation of NETs. We hypothesize that DNase I treatment only deconstructed the DNA back-bone, while remaining proteinases in the tissue space, such as the neutrophil elastase and myeloperoxidase could damage epithelial and endothelial cells or trigger them to express cytokines. Some previous research has confirmed that blocking NE and MPO or other components by using antibodies could alleviate cytotoxic effects of NETs in vitro [3, 26]. Apart from DNase I deconstructing NETs, their formation may actually depend on neutrophil elastase rather than myeloperoxidase [12]. Administration of neutrophil elastase inhibitor to NA mice blocks NET formation on neutrophils further preventing NETs or harmful components from being released into the extracellular space, thereby allowing NEi to alleviate inflammation caused by the NETs. These results suggest that NETs promote airway inflammation in the NA model independently of their structural integrity. Additionally, we also measured the lymphocyte and macrophage numbers under both NEi or DNAse treatments. A previous study has shown that NEi can reduce the number of various types of inflammatory cells in the airways of mice with eosinophilic asthma sensitized and stimulated by OVA, which may be related to the inhibition of early neutrophil inflammation [27]. Another study has shown that NETs can promote lymphocyte aggregation [28]. In our model, the number of airway lymphocytes decreased after the intervention of NEi and DNase, indicating that lymphocyte aggregation may be related to the generation of NETs and the integrity of the DNA skeleton structure. Macrophages are directly and indirectly linked to neutrophils and NETs. Mouse epithelial cells can secrete CCL2/3/4/5 and other mononuclear macrophage chemokines to recruit macrophages [29]. In macrophages, both pathogen-associated molecular patterns and damage-associated molecular patterns can enhance cell recruitment and promote inflammation [30]. NETs are effective monocyte stimulators to provide signals that upregulate endogenous damage-associated molecular patterns [31]. Acting as a negative feedback mechanism, NETs are internalized by extracellular DNase I and macrophages and then digested by TREX1 (also known as DNase III) [32]. In patients with severe asthma plus high extracellular DNA (these patients have high neutrophils in the airway), although the number of macrophages in the airway were increased, their proportion was decreased [33]. Additionally, a previous study found that phagocytosis of macrophages in patients with neutrophilic asthma is reduced [34]. In the present study, we found that the reduction of NETs resulted in the reduction of macrophages after NEi intervention; while under the intervention of DNase, there was no significant reduction. This shows that the number of macrophages is related to the production of NETs, and is independent of the integrity of the DNA skeleton structure. However, the effects and regulatory mechanisms of NETs on macrophages and lymphocytes need to be further studied.

Neutrophil chemotaxis is used by chemokines such as CXCL1, CXCL2, and CXCL8 [35]. A previous study has found that CXCL8 and IL-1β expression was promoted in both COPD and asthma patients [20]. Airway epithelial cells, HBEs, and HAEs are responsible for airway inflammation [3, 36]. When exposed to external environment, harmful particles, chemicals, or pathogens, these intrinsic cells act as the first line of defense, as they express cytokines and chemokines under direct stimulation by stimulants or indirect stimulation by pro-inflammatory substances [19]. CXCL1, CXCL2, and CXCL8 expression was enhanced in primary HBEs, primary bronchial fibroblasts, and human smooth muscle cells after IL-17A stimulation [37]. Additionally, the secretion of CXCL8/IL-8 and IL-6 was significantly enhanced in alveolar cells and HBEs after NET treatment [38]. In this study, the results confirmed that NET stimulation enhanced the secretion of CXCL1, CXCL2, and CXCL8 levels by HBEs and A549 cells which attracted neutrophils to migrate in vivo and in vitro. This result showed that NETs stimulate airway cells to secrete chemokines, leading to recruitment of the second wave of neutrophils.

TLRs are identified as key elements in the immune response, mainly responsible for regulating immune defense mechanisms against pathogenic microorganisms [39, 40]. TLR4 is the first recognized TLR among a total of 10 receptors, which can activate the NF-κB signaling pathway, thereby promoting transcription and translation of chemokines including CXCL1, CXCL2 and CXCL8 [41, 42]. A previous study found that inactivated NF-κB pathway alleviates the pathological changes in asthma [43]. Here, we confirmed that the TLR4/NF-κB signaling pathway was activated in the NA model and in the HBEs and A549 cells stimulated by NETs. Additionally, TLR4 specific inhibitor TAK-242 could silence the TLR4/NF-κB signaling pathway, relieve inflammatory response and airway resistance, reduce production of CXCL1, CXCL2, and CXCL8 chemokines, and inhibit the migration infiltration of neutrophils in vivo and in vitro. This result showed that NETs stimulate airway cells to secrete chemokines, resulting in the second wave of neutrophil recruitment by activating the TLR4/NF-κB signaling pathway.

Airway resistance is characteristic of asthma, in many clinic related studies, extracellular DNA, CXCL8, and neutrophils in BALF or induced sputum were negatively related to lung function and degree of symptom severity [14, 15, 20]. Nebulization of cystic fibrosis patients with DNase I could enhance their lung function and attenuate symptoms [7]. Other animal-based experiments have also shown that administration of DNase I could reduce airway hypersensitivity and oxidative stress in asthmatic mice [7]. However, there is evidence that DNase I could enhance NE activity in septum of CF patients, therefore, administrating DNase I to these patients would not always be effective as evidenced by the number of neutrophils in BALF not being related to airway hypersensitivity of asthmatic mice [7, 44]. The data obtained in this study shows lower airway resistance after administration of TLR4 inhibitor and NEi, but not after DNase I administration, which complies with variations in the degree of inflammation. However, we have still not found any direct evidence that NETs or neutrophils contribute to airway hypersensitivity. A complicated interaction of networks may be involved in airway smooth muscle constriction.

**Figure 7:**
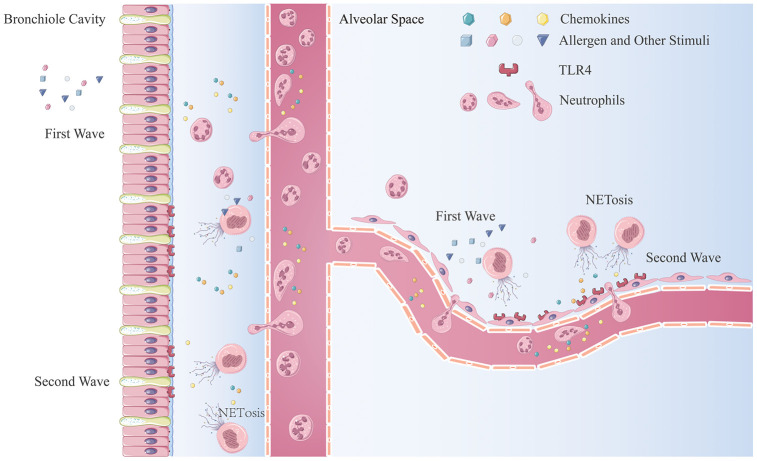
**The mechanism of neutrophilic inflammation amplification cycle initiated by NETs.** (**A**) Allergen or other stimuli cause the first wave of neutrophil recruitment. (**B**) NET formation are stimulated by inflammatory factors, bacterial components, or other stimulators. (**C**) NETs aggregate at the bronchiole and lung-bubble cavities and stimulation of the HBEs and HAEs expressing neutrophilic chemokines. (**D**) Neutrophils migrate from the peripheral blood to lung interstitial tissue and parenchyma generate more NETs when stimulated.

In this study, we demonstrated an amplification cycle of neutrophil recruitment (Figure 7). The first wave of neutrophils is recruited by an early inflammatory response resulting from stimulation of allergens or irritants. Thereafter, NET production results in the death of neutrophils releasing NETs into the extracellular space. Airway epithelial cells irritated by NETs and HAEs release chemokines, CXCL1/2/8, via activation of the TLR4/NF-κB pathway and recruitment of more neutrophils to the inflammatory sites. In the inflammatory environment, newly recruited neutrophils are activated and undergo NET production to further enhance the inflammatory response.

In conclusion, this study revealed that NETs could trigger airway epithelial cells and HAEs to express chemokines which recruit more neutrophils via activation of the TLR4/NF-κB pathway in NA mice. Further detailed understanding of the mechanism of NET formation during neutrophilic inflammation in asthma could offer new potential therapeutic strategies for NA and other neutrophilic inflammatory diseases.

## MATERIALS AND METHODS

### Mouse model of NA

Wild type male C57BL6 mice were purchased from the Experimental Animal Center of Central South University (Changsha, China). All mice were aged 6-8 weeks and weighed between 18 g and 22 g. All mice were kept in specific pathogen-free environment with freely available standard food and water. All animal experiments were approved by the Animal Care and Use Committee of Central South University and were conducted in accordance with the National Institutes of Health Guidelines for the Care and Use of Laboratory Animals. The LPS/OVA administration into the NA mouse model was performed in accordance with Wilson’s protocol [44]. Briefly, on day 0 and day 6, mice were anesthetized by pentobarbital administration and were administered 100 μg low-endotoxin ovalbumin (OVA, Grade V, Sigma-Aldrich, St. Louis, MO, USA) and 15 μg LPS (Sigma-Aldrich) dissolved in 50 μl PBS (delivery vehicle) intratracheally (i.t.), followed by 200 μl of air, on day 13, all mice were challenged with a single nebulization comprising of an aerosol of 1% OVA (Grade III, Sigma-Aldrich) in saline for 40 minutes.

### In vivo DNase I treatment

Mice were administered 3 mg/ml DNase I (5 mg/kg, Roche, USA) or vehicle control (8.77 mg.ml NaCl, 0.15 mg/ml CaCl_2_ DNase I working solution) i.t. right before and 6 hours after OVA challenging.

### In vivo treatment with neutrophil elastase inhibitor (NEi) and TLR4 inhibitor

In vivo NE and TLR4 inhibition were accomplished by intraperitoneally injecting 10 mg/kg of an NEi, sivelestat sodium tetrahydrate (Selleck, USA) every day for 4 days before harvest and a TLR4 inhibitor, TAK-242 (Selleck, USA) right before nebulization and 6 hours after nebulization. The corresponding control group received an equivalent PBS dose.

### Hematoxylin-eosin staining

For histopathological analysis, the right lung lobes were fixed in 4% PFA and embedded in paraffin. Sections (4 μm) were placed onto glass slides and stained with hematoxylin and eosin (HE).

### Quantification of extracted double-stranded DNA and total protein

Double-stranded DNA extracted from the BALF supernatant was quantified by a Quant-iT PicoGreen assay kit in accordance with the manufacturer’s instructions (Invitrogen, Burlington, ON, Canada). The dsDNA extracted from NETs of treated cells were quantified using a Nanodrop2000 spectrophotometer (ThermoFisher Scientific). The total protein in the BALF supernatant was quantified using an Enhanced BCA Protein Assay Kit (Beyotime, Beijing, China), following the manufacturer’s protocol.

### Western blotting

Protein detection for whole cell lysates of mice lung tissue and in vitro cultured cells was conducted using western blotting. PVDF membranes were incubated overnight with Cit-H3 (1:500, Abcam, USA) and NF-κB (1:500, ABclonal Technology, China) antibodies. Anti-GAPDH antibody (1:5000, Proteintech, USA) was used as the internal control.

### Immunohistochemistry and immunofluorescence

Immunohistochemistry was used to detect CXCL1, CXCL2, and CXCL8 levels in airway cells of the mouse model. Immunofluorescence was used to detect Cit-h3 (Abcam) and MPO (Abcam) expression in vivo and in vitro. Paraffin-embedded mouse lungs were sectioned at 4 μm thickness and mounted on anti-off slides. After dewaxing and hydration, slides were immersed for 20 minutes in boiling Tris-EDTA antigen retrieval solution (pH9.0, ServiceBio, China). Antigen blocking was performed by coating the slides with 10% normal serum and incubating for 1 hour at room temperature followed by incubation with primary antibody overnight at 4°C for antigen detection. Immunohistochemistry was performed using a 2-step detection kit (ZSGB-Bio, China) for DAB reaction, in accordance with the manufacturer’s instructions. Immunofluorescence was performed using Alexa Fluor 488 donkey anti-rabbit (Abcam) and Alexa Fluor 647 donkey anti-goat (Abcam) as secondary antibodies and incubating for 1 hour at room temperature to detect primary antibodies. Fluoroshield mounting media with DAPI (Abcam) was used for mounting and DNA detection. The protocol for detection of NETs in PMA stimulated neutrophils was similar for the paraffin-embedded slides, but the samples on carry sheet glass were fixed by methyl alcohol for 20 minutes without the dewaxing and the hydration processes.

### Enzyme-linked immunosorbent assay (ELISA)

CXCL1, CXCL2, and CXCL8 levels in mouse BALF supernatant were measured using commercial mouse enzyme-linked immunosorbent assay (ELISA) kits (RayBiotech for CXCL1 and CXCL2, USA; Neobioscience for CXCL8, China). The assays were performed following the manufacturer’s instructions.

### In vitro generation of neutrophils and NETs

Polymorphonuclear neutrophils (PMNs) were obtained by performing density gradient centrifugation. Briefly, whole blood was diluted 1:1 diluted in sterilized PBS, loaded on a human lymphocyte separating media followed by density gradient centrifugation (800 *g*, 21°C, without break), leaving RBCs and PMNs at the bottom of the centrifugation tube, which were then mixed with 6% dextran 40. Following 30 minutes of sedimentation, the supernatant was centrifugated at 1000 *g* for 5 minutes. Red blood cell lysing solution (Geneview, China) was used to resuspend the neutrophilic rich pellet. This was then recentrifuged and resuspended in RPMI1640 culture media with 10% FBS. The purity of the neutrophils obtained (consistently > 95%) was verified by Giemsa staining, and cell viability (> 95%) was verified by trypan blue staining. PMNs were counted and loaded into 6-well cell culture plates (10^6^/well). Phorbol 12-myristate 13-Acetate (PMA, Sigma-Aldrich) at 500 nM was added to the PMNs and incubated for 4 hours at 37°C with 5% CO_2_. After incubation, the supernatant was aspirated by gently washing the wells with cold PBS and all the PBS solution was collected. This solution was then centrifuged at 400 *g* at 4°C to separate the remaining neutrophils, and the supernatant was divided into 1.5 ml micro-centrifuge tubes and centrifuged at 18000 *g* at 4°C. The pellet was resuspended using cold PBS and the DNA concentration was adjusted for subsequent experiments. 24-well cell culture plates and carry sheet glass was used for NETs detection by immunofluorescence.

### Airway resistance

After anesthetizing with pentobarbital (125 mg/kg i.p.), the mice were intubated and connected to a modular and invasive airway resistance detection system (Buxco). Mice were ventilated and a steady baseline of airway pressure was generated. Next, graded doses of methacholine (0, 3, 6, 12, 25 mg/ml, Sigma-Aldrich) were administered to mice via a nebulizer, and the changes in airway resistance were recorded.

### Cells and treatments

HBEs and HAEs (A549) were obtained from cell repository of Advanced Research Center, Central South University, and were cultured in Dulbecco’s modified Eagle’s medium (DMEM) or Roswell Park Memorial Institute 1640 (RPMI1640) with 10% FBS and 1% penicillin-streptomycin. The cells were treated with human-derived NETs (DNA concentration at 5 μg/ml), TAK-242 (3 μM/L, Selleck USA), or DNase I (3 mg/ml, ThermoFisher) for 24 h after the cells achieved a growth confluence of 80%.

### Quantitative PCR analysis

Total RNA was extracted from matured cells using RNeasy Mini Kit (Qiagen, Germany) while cDNA was synthesized using PrimeScript RT reagent Kit with gDNA Eraser (Takara, Japan). Quantitative real-time polymerase chain reaction (RT-qPCR) was performed with a total volume of 10 μl by using specific primers (Table 1) and TB Green Premix Ex Taq II kit (Takara, Japan) in the ViiA™ 7 Real-Time PCR System (Applied Biosystems, USA). All analyses were performed following the manufacturer’s instructions.

**Table 1:** RT-qPCR primers.

**Primers**	**Sequence (5′ to 3′)**
CXCL1 Forward	AGTGTGAACGTGAAGTCCCC
CXCL1 Reverse	ATGGGGGATGCAGGATTGAG
CXCL2 Forward	CCTGCAGGGAATTCACCTCA
CXCL2 Reverse	TGAGACAAGCTTTCTGCCCA
CXCL8 Forward	CTCCAAACCTTTCCACCCCA
CXCL8 Reverse	TTCTCCACAACCCTCTGCAC
GAPDH Forward	TCGGAGTCAACGGATTTGGT
GAPDH Reverse	TGGAATTTGCCATGGGTGGA

### Migration assay

Cell culture supernatant was collected for the transwell migration assay. Fresh human neutrophils were separated as per the previously mentioned method. Transwell plates were incubated in RPMI1640 culture media for 1 hour at 37°C before loading the cells. Neutrophils were resuspended in 10^5^/ml of media and 10^4^ cells were loaded into the upper chamber of the transwell systems while 600 μl cell culture supernatant was loaded into the lower chamber of the transwell systems. The lower chamber was exposed to 0.1% crystal violet and methyl alcohol for 30 minutes for fixing and staining.

Additionally, neutralizing antibodies were added to the lower chamber right before incubation to neutralize CXCL1 (1:200, Arigo, China), CXCL2 (1:500, GeneTex, USA) and CXCL8 (1:100, Arigo, China).

### Detection of cytokine concentration using Luminex assays

Luminex assays were performed in accordance with the instruction provided in the kit (LXSAHM-03, R&D systems, USA) and the instrument (X-200, Luminex, USA). The concentration was detected by Univ bio-technology corporation (China).

### Statistical analyses

All data were recorded as the mean ± standard deviation (SD). Differences between more than two sets of data were evaluated by one-way ANOVA and Tukey’s multiple-comparisons test. P < 0.05 (two-tailed) was considered statistically significant.

## Supplementary Material

Supplementary Figure 1
