# Correction for: Neutrophil extracellular traps amplify neutrophil recruitment and inflammation in neutrophilic asthma by stimulating the airway epithelial cells to activate the TLR4/ NF-κB pathway and secrete chemokines

**DOI:** 10.18632/aging.205770

**Published:** 2024-04-30

**Authors:** Rongjun Wan, Juan Jiang, Chengping Hu, Xi Chen, Cen Chen, Bingrong Zhao, Xinyue Hu, Zhiyuan Zheng, Yuanyuan Li

**Affiliations:** 1 Department of Respiratory Medicine, National Key Clinical Specialty, Branch of National Clinical Research Center for Respiratory Disease, Xiangya Hospital, Central South University, Changsha, Hunan, China; 2 Hunan Provincial Clinical Research Center for Respiratory Diseases, Xiangya Hospital, Changsha, Hunan, China; 3 National Clinical Research Center for Geriatric Disorders, Xiangya Hospital, Changsha, Hunan, China

**Keywords:** neutrophilic asthma, neutrophil extracellular traps, chemotaxis, airway inflammation

**This article has been corrected:** The authors found that in **Figure 3B** the HE staining image intended for the TAK-242 group was inadvertently replaced with one from the DNase group in **Figure 2B**, leading to a duplication. They also found duplications in **Figure 3J**, where IHC images of CXCL2 and CXCL8 expression in the control group were mistakenly used for the TAK-242 group. The authors corrected these errors with images from the original sets of experiments. These errors do not affect the study’s conclusions, because the analysis was conducted using the correct datasets from the repeated experiments.

The corrected version of **Figure 3** is provided below.

**Figure 3:**
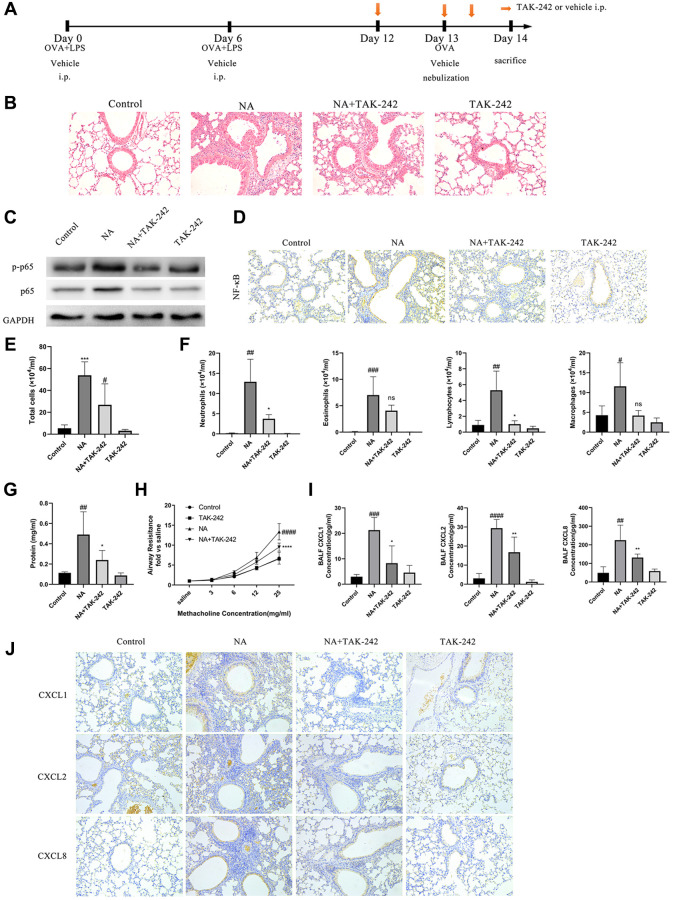
**Blocking the TLR4/NF-κB pathway with TAK-242 reduced the expression of chemotactic factors and alleviate airway inflammation in NA mice.** (**A**) Flow chart for intraperitoneal administration of TAK-242. (**B**) HE staining results showed that TAK-242 treatment could alleviate inflammation (200X). (**C**) p65 NF-κB and p-p65 NF-κB expression was measured by western blotting. (**D**) p65 NF-κB expression was measured by immunohistochemistry (200X). (**E**–**G**) Total cells, differential cells, and total protein were measured in the BALF of NA mice. (**H**) Airway resistance was measured after methacholine treatment. (**I**) CXCL1, CXCL2, and CXCL8 expression in the BALF of NA mice was measured by ELISA. (**J**) CXCL1, CXCL2, and CXCL8 expression was measured by Immunohistochemistry in the lung tissues of NA mice. ^#^*P* < 0.05, ^##^*P* < 0.01, ^###^*P* < 0.001, ^####^*P* < 0.0001 vs. control group. ^*^P < 0.05, ^**^P < 0.01: ^***^P < 0.001, ^****^P < 0.0001 vs. NA group. ns = no statistical difference vs. NA group.

