# Supplementary material for: Neutrophil extracellular traps amplify neutrophil recruitment and inflammation in neutrophilic asthma by stimulating the airway epithelial cells to activate the TLR4/ NF-κB pathway and secrete chemokines
Source: Aging (Albany NY). 2020 Aug 5;12(17):16820–36. doi: 10.18632/aging.103479 (PMC7521522; doi:10.18632/aging.103479)
Supplement: Supplementary Figure 1 [file aging-12-103479-s001..pdf]

SUPPLEMENTARY FIGURE

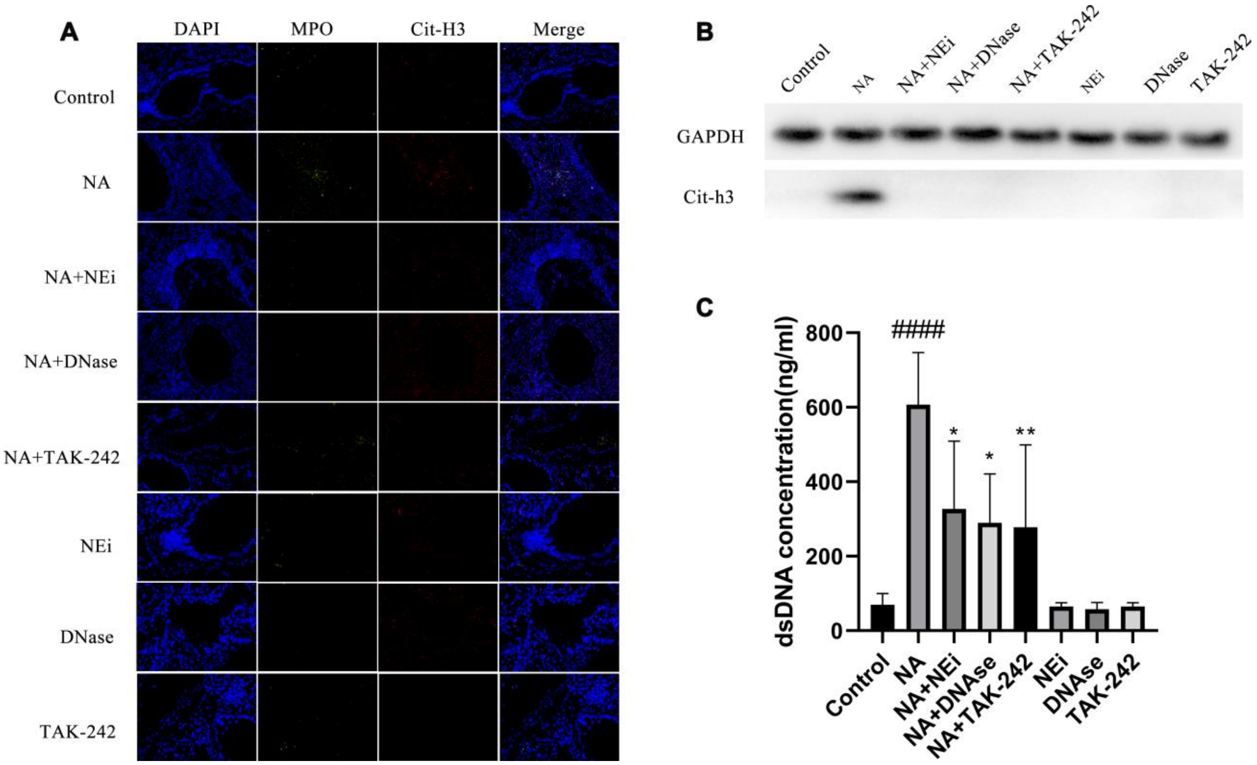

Supplementary Figure 1. NETs were detected in NA mice and could be removed by NE inhibition, DNase I degeneration, and TLR4 blocking. (**A**–**C**) Assessment of NETs by immunofluorescence, western blot, and PicoGreen. (**A**) DNA was stained by DAPI (blue), MPO and Cit-H3 were labeled by fluorescent secondary antibodies (red and green); pictures were captured at 200X. (**B**) Western blot analysis of Cit-H3. (**C**) BALF dsDNA concentration detected by PicoGreen analysis. NET generation was detected in NA mice, and could be blocked by NEi, DNase I, and TAK-242 administration. ####: P<0.0001 vs control group. *: P<0.05, **: P<0.01 vs NA group.
